# Focusing on cyclin-dependent kinases 5: A potential target for neurological disorders

**DOI:** 10.3389/fnmol.2022.1030639

**Published:** 2022-11-10

**Authors:** Zhen Tian, Bin Feng, Xing-Qin Wang, Jiao Tian

**Affiliations:** ^1^College of Pharmaceutical Sciences, Southwest University, Chongqing, China; ^2^State Key Laboratory of Military Stomatology, National Clinical Research Center for Oral Diseases, Shaanxi International Joint Research Center for Oral Diseases, Department of Pharmacy, School of Stomatology, Fourth Military Medical University, Xi’an, China; ^3^Department of Neurosurgery, Nanfang Hospital, Southern Medical University, Guangzhou, China; ^4^Department of Infection, Children’s Hospital of Chongqing Medical University, National Clinical Research Center for Child Health and Disorders, Ministry of Education Key Laboratory of Child Development and Disorders, Chongqing Key Laboratory of Child Infection and Immunity, The First Batch of Key Disciplines On Public Health in Chongqing, Chongqing, China

**Keywords:** Cdk5, p35, neurological disorders, Parkinson’s disease, Alzheimer’s disease, stroke

## Abstract

Cyclin-dependent kinases 5 (Cdk5) is a special member of proline-directed serine threonine kinase family. Unlike other Cdks, Cdk5 is not directly involved in cell cycle regulation but plays important roles in nervous system functions. Under physiological conditions, the activity of Cdk5 is tightly controlled by p35 or p39, which are specific activators of Cdk5 and highly expressed in post-mitotic neurons. However, they will be cleaved into the corresponding truncated forms namely p25 and p29 under pathological conditions, such as neurodegenerative diseases and neurotoxic insults. The binding to truncated co-activators results in aberrant Cdk5 activity and contributes to the initiation and progression of multiple neurological disorders through affecting the down-stream targets. Although Cdk5 kinase activity is mainly regulated through combining with co-activators, it is not the only way. Post-translational modifications of Cdk5 including phosphorylation, S-nitrosylation, sumoylation, and acetylation can also affect its kinase activity and then participate in physiological and pathological processes of nervous system. In this review, we focus on the regulatory mechanisms of Cdk5 and its roles in a series of common neurological disorders such as neurodegenerative diseases, stroke, anxiety/depression, pathological pain and epilepsy.

## Introduction

Cyclin-dependent kinases (Cdks) are a group of small proline-directed serine/threonine kinases mainly involved in the modulation of cell cycle, gene transcription and cell differentiation (Malumbres, 2014; Xie et al., 2022). Cyclin dependent kinase 1 (Cdk1) was the first discovered Cdk, which was initially named as cell division cycle 2 (Cdc2) because of its high homology with a yeast fission kinase of the same name (Lee and Nurse, 1987). Until now, twenty-one genes encoding Cdks and five genes encoding Cdk-like kinases have been found in human genome (Malumbres et al., 2009). Generally, Cdks are activated through associating with specific cyclin regulatory subunits and regulated through phosphorylation of specific T-loops by Cdk-activating kinase (Malumbres et al., 2009). Among the family of Cdks, Cdk5 is a special one though it shares significant sequence homology with other members (Hellmich et al., 1992). It can neither be activated by cyclins nor involved in cell division directly. Cdk5 is ubiquitous in kinds of tissues, but its activity is extremely high in post-mitotic neurons because its activators p35 and p39 are selectively expressed in these cells (Tsai et al., 1994; Ko et al., 2001). P35 and p39 are different from cyclins as they lack the typically conserved amino acid sequences. The expression of Cdk5 and p35/p39 transcripts overlaps spatially and temporally in the developing brain, and upon directly binding, they form active complexes and induces the alterations of downstream signal pathways.

In the developing nervous system, Cdk5 seems to be expressed mainly in terminally differentiated neurons but not proliferating cells. Because the expression and kinase activity of Cdk5 was shown to increase progressively from embryonic day 15 (E15) and reaches the peak at E17 in developing mouse forebrain, which period is a critical time widow for neuronal differentiation (Tsai et al., 1993). During the embryonic brain development of rats, the expression pattern of Cdk5 is similar to that of mouse, with a gradual increase from E12 to E18 and reaches a peak at postnatal day 7 (P7), which is then maintained at this level through adulthood and aged phase (up to 18 months old) (Wu et al., 2000). In the adult central nervous system, Cdk5 is distributed throughout the brain and spinal cord, especially in the hippocampus, cerebellum, cerebral cortex, olfactory bulb, amygdala and brain stem. Generally, the level of Cdk5 is higher in the large-sized and medium-sized neurons such as hippocampal and cortical pyramidal cells, cerebellar Purkinje cells, motoneurons in spinal cord (Ino et al., 1994). The expression level of Cdk5 in various brain regions was also compared and quantified. The results showed that there was no obvious difference about Cdk5 level in the cerebral cortex, hippocampus, cerebellum and striatum of rats aged from postnatal day 14 (P14) to 18 months old (18 M) (Wu et al., 2000).

As for p35, the co-activator of Cdk5, its expression becomes detectable in E16 rat brains and gradually increases to its peak level at P7–P14, then declines in the adult and aged rat brains. The Cdk5 kinase activity is low at E12 and it also progressively reaches a peak between P7 and P14, and then declines from 1 to 3 M, a timetable correlating well with the expression pattern of p35 (Wu et al., 2000). Different from the expression pattern of Cdk5 protein, p35 expression and Cdk5 kinase activity were persistently higher in the hippocampus and cerebral cortex than cerebellum or striatum in rats from P14 to 18 M, indicating that Cdk5/p35 complex may have region-specific functions (Wu et al., 2000). P39 is the isoform of p35 with over 50% homology in genomic sequence, the functions of p39 largely overlap with those of p35. However, their spatial and temporal expression patterns are different, with p35 being highly expressed from embryonic to postnatal stage while p39 is scarcely expressed until postnatal (Tsai et al., 1994; Ko et al., 2001). In addition, the expression of p35 is mostly prominent in cerebral cortex while p39 is primarily expressed in brain stem, cerebellum and spinal cord (Ko et al., 2001). Furthermore, the binding affinity of p39 with Cdk5 is lower whereas the Cdk5-p39 complex is more stable compared with Cdk5-p35 (Yamada et al., 2007; Minegishi et al., 2010).

Cyclin-dependent kinases 5 complex plays a very important role in neuronal functions from embryogenesis to postnatal brain modulation. During embryogenesis, Cdk5 is essential for normal brain development and conditional deletion of Cdk5 is lethal (Ohshima et al., 1996; Shah and Lahiri, 2014). It is because that Cdk5 governs multiple steps of cortical neuronal migration, including the formation of multipolar processes (Kawauchi et al., 2006), gain of neuronal polarity (Ohshima et al., 2007) and the locomotion mode (Nishimura et al., 2010). Cdk5 deficiency will lead to the lack of cerebral cortical laminar structure and cerebellar foliation in mice (Ohshima et al., 1996). Mice lacking p35 show less severe corticogenesis defect and only suffer from sporadic adult lethality, which may be attributed to functional compensation by another Cdk5 activator, p39 (Chae et al., 1997). Although loss of 39 is not lethal, it attenuates overall Cdk5 activity in neurons as well as leads to aberrant axonal growth and impaired dendritic spine formation through affecting Cdk5 targets governing neuronal differentiation and network formation (Li et al., 2016). In adult brain, emerging evidences have indicated that Cdk5 complex regulated multiple neuronal functions including neuronal survival, neurite and axon outgrowth, synaptic plasticity, neurotransmission (Ye et al., 2014; Sasamoto et al., 2017; Takahashi et al., 2022). Because of its extensive and crucial roles in nervous system, the dysfunction of Cdk5 is critically involved in numerous neurological disorders such as neurodegenerative diseases, stroke, psychiatric disorders, pathological pain, epilepsy and so on. The multifaceted roles of Cdk5 make it to be an attractive target for therapeutic intervention. An updated review on the relationship between Cdk5 and nervous system dysfunction can help us better understand its potential clinical value. Therefore, we comprehensively summarized the role of Cdk5 in a series of common neurological disorders in this paper. The synthesis of these scientific evidences about Cdk5 could facilitate future research to further explore the potentiality of drugs targeting Cdk5 for clinical therapy.

## Regulation of cyclin-dependent kinases 5 kinase activity

The aberrant Cdk5 activity is critically involved in the pathogenesis of neurological disorders, so the regulation of kinase activity may be a potential strategy for disease treatment. Cdk5 kinase activity is mainly regulated by the available protein amounts of p35/p39 and their truncated fragments. p35 and p39 levels are determined by the balance between synthesis and degradation, which are cleavage by the ubiquitin-proteasome system with a half-life of about 30 and 120 min for p35 and p39, respectively (Minegishi et al., 2010). Calpain, a Ca^2+^ activated protease, is involved in multiple physiological processes and plays an important role in maintaining normal neuronal function. The activation of calpain depends on Ca^2+^ concentration, which makes it vulnerable to changes of Ca^2+^ homeostasis (Metwally et al., 2021). Following neurotoxic insults, sustained Ca^2+^ elevation leads to the over-activation of calpain, which in turn cleaved p35 and p39 into N-terminal p10 and C-terminal p25 or p29 truncated fragments (Lee et al., 2000). However, the half-life of truncated protein fragment is much longer than that of mother molecule and it can form a more stable complex with Cdk5, resulting in the prolonged activation of Cdk5 (Patrick et al., 1999; Minegishi et al., 2010). In addition, the N-terminal p10 region of p39 and p35 contains a second Gly for myristoylation and Lys clusters, the localization motifs helping to anchor the active complex at membrane. However, the truncated forms derived from cleavage of p35 or p39 lost the myristoylation signal that normally maintains Cdk5 at the membrane, leading to accumulation of the complex in nuclear and perinuclear regions (Asada et al., 2008). This aberrant subcellular localization of Cdk5/co-activator complex disturbs its substrate specificity and makes it target not only physiological substrates but also non-physiological substrates, resulting in neurotoxicity and cell death (Mushtaq et al., 2016). Thus, the hyperactivation and mislocalization of Cdk5 caused by p25 accumulation lead to the dysfunction of Cdk5, which contributes to the pathogenesis of various neurological diseases.

Although binding with the regulatory subunits is sufficient to activate Cdk5, its kinase activity can also be regulated by other pathways, such as phosphorylation, S-nitrosylation, sumoylation, and acetylation. Thr14, Tyr15, and Ser159 are the three phosphorylation sites of Cdk5 found so far. Phosphorylation at the sites of Thr14 and Ser159 produces opposite outcomes on Cdk5 activity, with the former inhibiting Cdk5 activity and the latter increasing its activity (Lee et al., 2014). The effect of phosphorylation at Tyr15 on kinase activity is still controversial. Some studies found that Cdk5 activity was enhanced by phosphorylation at Tyr15 while other study got the negative results. For example, Sasaki et al. (2002) reported that phosphorylation of Tyr15 by a non-receptor Src family tyrosine kinase Fyn increased Cdk5 enzymatic activity. However, Kobayashi et al. (2014) found that phosphorylation of Tyr15 had no effect on the activation of Cdk5. Thus, future studies are still needed to further clarify the function of Tyr15 phosphorylation as it is helpful for uncovering new regulatory pathway of Cdk5 activity.

Besides phosphorylation, the S-nitrosylation of Cdk5 is another mechanism to regulate kinase activity. It has been shown that the S-nitrosylation at cysteine residues (Cys) 83 and 157 contributed to the elevation of Cdk5 kinase activity (Qu et al., 2011, 2012). The S-nitrosylation of Cdk5 will cause transnitrosylation, a process of transferring NO group to dynamin-related protein 1 (Drp1), a protein controlling the process of mitochondrial fission (Westermann, 2010). S-nitrosylated Drp1 then stimulates excessive mitochondrial fission and damages the synapses, resulting in dendritic spine loss and synaptic failure finally (Cho et al., 2009). However, it is worth noting that the effect of S-nitrosylation at Cys83 on Cdk5 activity may be determined by the levels of exogenous NO. Cys83 S-nitrosylation by very high levels (non-physiological level) of NO could conversely inhibit Cdk5 activity though the concentrations of NO donors are never reached *in vivo* (Zhang et al., 2010). The cysteine residues of p35 can also be S-nitrosylated, it was found that S-nitrosylation of p35 at Cys92 by NO signaling led to its ubiquitination and degradation, which resulted in the reduction of Cdk5 activity (Zhang et al., 2015).

Sumoylation and acetylation are two other ways of post-translational modifications of Cdk5 activity. It has been shown that p35 is a novel sumoylation target and the sumoylation of p35 can enhance the activity of Cdk5/p35 complex (Buchner et al., 2015). However, it is still unclear whether Cdk5 can be sumoylated directly. With regard to acetylation, Cdk5 can be acetylated at the lysine residue site 33 (K33), a residue comprising the ATP binding pocket. The acetylation of Cdk5 at K33 causes an impairment of kinase activity due to the loss of ATP binding ability. The Cdk5 acetylation is negatively regulated by Sirtuin-1 (SIRT1), inhibition of SIRT1 will enhance nuclear Cdk5 acetylation whereas SIRT1 activation results in deacetylation of nuclear Cdk5 (Lee et al., 2018). Whether there are any other acetylation sites remains unclear, and the role of Cdk5 acetylation in neurological diseases is also still needed to be further investigated.

## Cyclin-dependent kinases 5: A culprit in neurological disorders

### Cyclin-dependent kinases 5 dysfunction triggers Parkinson’s disease

Parkinson’s disease (PD) is a common neurodegenerative movement disorder characterized by widespread degeneration of dopaminergic neurons located in the substantia nigra pars compacta (SNpc) and subsequent loss of dopamine reaching striatal projecting neurons (Obeso et al., 2010). The formation of intra-neuronal inclusions called Lewy bodies (LB) is a pathologic hallmark of PD and often correlates with the degree of cognitive decline (Schneider et al., 2012). Cdk5 and p35 has been found to be localized in LB in the SNpc of postmortem patients brains with PD (Nakamura et al., 1997). There is also sustained calpain-dependent conversion of p35 to p25 and enhanced Cdk5 activity in the brain of PD patients and animal models (Smith et al., 2003; Alvira et al., 2008). Quantitative phosphoproteomic analysis of α-synuclein transgenic mice showed that elevated Cdk5/p25 pathway activity contributed to SNpc dopaminergic neuronal death in model mice (Stillwell and Myers, 1988), which was also observed in mice administrated with 1-methyl-4-phenyl-1, 2, 3, 6-tetrahydropyridine (MPTP), a toxin used to induce PD model through destroying the nigrostriatal dopaminergic pathway (Binukumar et al., 2015). TFP5, a specific inhibitory peptide of Cdk5/p25 complex, suppressed the dopaminergic neuronal loss in SNpc and striatum, improved motor function of PD mice (Binukumar et al., 2015).

The mitochondrial dysfunction and subsequent oxidative stress are important mechanisms underlying the pathogenesis of PD. Abnormal Cdk5 activity contributes to mitochondrial dysfunction in PD by aberrant modulation of several key proteins involved in mitochondrial function. Drp1 is a GTPase that controls the process of mitochondrial fission. Drp1 is translocalized from the cytosol to the mitochondrial outer membrane (MOM) and then assembled into ring-like structures wrapping around MOM. Following GTP hydrolysis, Drp1 incises the membrane and instigates mitochondrial fission (Westermann, 2010). In non-human primate PD model, Cdk5 hyperactivity was shown to increase the phosphorylation of Drp1 at Ser616, which in turn increased GTPase activity and accelerated mitochondrial fission, ultimately induced dopaminergic neuronal loss in the SNpc (Park et al., 2019). Inhibition of Cdk5 activity prevented Drp1-dependent mitochondrial fission and neuron death following MPP+ treatment (Yang et al., 2020). Dysfunction of E3 ubiquitin ligases has a close relationship with mitochondrial defects and disruption of protein degradation. Parkin is an E3 ubiquitin ligase and plays an important role in maintaining mitochondrial function. It is able to prevent cell death from toxicity elicited by diverse insults (Petrucelli et al., 2002). However, the aggregation of insoluble Parkin will inhibit its catalytic activity and lead to dopaminergic neuron death caused by accumulation of toxic Parkin substrates, such as p38 (Avraham et al., 2007), ultimately contributing to the pathogenesis of PD (Rubio de la Torre et al., 2009). Cdk5 was demonstrated to phosphorylate Parkin at Ser131 both *in vitro* and *in vivo*, decreased its solubility, induced its accumulation and subsequently reduced E3 ubiquitin-ligase activity (Avraham et al., 2007). Consistent with this, enhanced p25 level and elevated Parkin phosphorylation were also found in several brain areas of PD patients (Rubio de la Torre et al., 2009). Glycoprotein 78 (GP78), another E3 ubiquitin ligase, is involved in regulating mitochondrial function. Cdk5 was found to directly phosphorylate GP78 at Ser516, which promoted its ubiquitination and degradation, ultimately caused neuronal death in both cellular and animal PD models (Wang et al., 2018).

Mitochondrial dysfunction and aggregation of damaged mitochondria can cause remarkable oxidative stress indicating by excessive accumulation of cellular reactive oxygen species (ROS). Peroxidases are a series of antioxidant enzymes with the capacity to catalyze hydrogen peroxide into stable non-toxic molecules. Cdk5 was demonstrated to phosphorylate peroxidases 2 (Prx2) at Thr89 and this phosphorylation decreased its peroxidase activity and induced ROS over-generation and dopaminergic neuron loss in the SNc following MPTP insult (Qu et al., 2007). Increased Prx2 phosphorylation in nigral neurons was also observed in brain tissue of postmortem PD patients. Interestingly, the insults-induced cleavage of p35 could also generate p10, a pro-survival N-terminal domain of p35 with the ability of preventing Cdk5/p25-mediated Prx2 phosphorylation and ROS accumulation (Zhang L. et al., 2012). Neuroinflammation is another outcome of mitochondrial dysfunction. Cdk5-mediated inflammasomes activation was observed in the SNpc of PD mouse model and in cerebrospinal fluid of PD patients. Inhibition or deletion of Cdk5 both could inhibit the inflammasome activation and delay the progression of PD in animal models (Zhang P. et al., 2016).

Excessive autophagy is closely related with neuronal death and there is over-production of autophagy markers in PD brains (Anglade et al., 1997). Knockdown of Cdk5 inhibited autophagy-mediated α-synuclein aggregation and promoted the functional recovery in PD mice (Su et al., 2015). Endophilin B1 (EndoB1) has been reported to be implicated in autophagy induction (Takahashi et al., 2007). In MPTP-induced PD model, elevated Cdk5 activity led to EndoB1 phosphorylation at Thr145 and then promoted EndoB1 dimerization, beclin-1 recruitment and autophagy induction, resulting in neuronal loss. EndoB1 or Cdk5 knockdown both remarkably attenuated the neuronal death in PD through inhibiting aberrant autophagy (Wong et al., 2011). Besides autophagy, several other cell death pathways mediated by Cdk5 were also shown to be involved in PD. Myocyte enhancer factor 2 (MEF2), one member of transcription factors family, is an endpoint for diverse signaling pathways that control cellular survival and apoptosis (Mao et al., 1999). Abnormal Cdk5/MEF2 signaling pathway contributes to dopaminergic neuronal death. Upon MPTP insult, the hyperactivity of Cdk5/p25 inactivated MEF2 and led to dopaminergic neuronal loss in the SNpc and formation of LB. Inhibition of Cdk5/p25 complex with TFP5 decreased the levels of MEF2 inactive form, thus significantly suppressed dopaminergic neuronal death (Zhang Q. et al., 2016). Nur77 is one important downstream survival effector of MEF2. Nur77 deficient mice are more sensitive to dopaminergic neuronal loss and exhibit more serious nigrostriatal damage in response to MPTP treatment (Mount et al., 2013). Cdk5-mediated MEF2 phosphorylation led to its inactivation and subsequently resulted in markedly Nur77 reduction in the nigrostriatal region following MPTP injection (Mount et al., 2013). These results imply that Cdk5-MEF2-Nur77 pathway is involved in the dopaminergic neuronal loss. Raf kinase inhibitor protein (RKIP) is able to block Ras/Raf/MEK/ERK signaling pathway by inhibiting Raf1-mediated MEK1 phosphorylation (Yeung et al., 1999). Downregulation of RKIP will lead to over-activation of ERK/MAPK pathway, which is resulted from losing control of Raf-1 (Granovsky and Rosner, 2008). Cdk5 was reported to phosphorylate RKIP at Thr42 and promote the degradation of RKIP. In PD animal models and post-mortal PD patients, Cdk5-mediated RKIP phosphorylation and degradation were demonstrated to occur with excessive activation of the ERK/MAPK cascade followed by cell cycle re-entry and neuron death (Wen et al., 2014).

**FIGURE 1:**
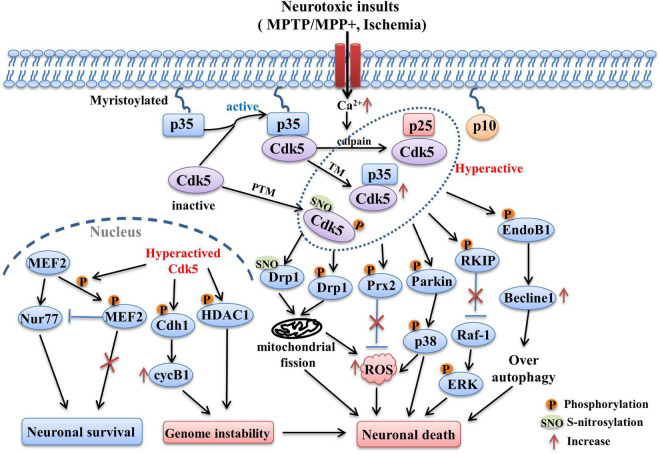
The Cdk5-related pathways involved in PD and stroke. Following neurotoxic insults, sustained Ca^2+^ elevation induces the over-activation of calpain, which in turn cleaves p35 into p25 and p10 fragments. The half-life of p25 is much longer than p35, it can form more stable complex with Cdk5 and lead to abnormally elevated Cdk5 activity. Its activity can also be regulated by post-translational modulations (PTM). The phosphorylation and S-nitrosylation of certain specific sites are able to hyperactive Cdk5 (e.g., phosphorylation at Ser159, S-nitrosylation at Cys83 and Cys157). In addition, transcriptional modulation (TM) of Cdk5 and p35 can also increase their expression and activity, which is mediated by some related transcription factors (e.g., Fos, CREB). Hyper-activation of Cdk5 then triggers the phosphorylation of multiple substrates including EndoB1, RKIP, Parkin, Prx2, Drp1, MEF2, Cdh1, and HDAC1. Changes in the phosphorylation state of these key substrates induced the occurrence of detrimental events such as mitochondrial dysfunction, oxidative stress, over-autophagy, which in turn contribute to the neuronal loss in PD and stroke.

The pathways related with Cdk5 in PD are summarized and presented as Figure 1.

### Cyclin-dependent kinases 5 facilitates the initiation and progression of Alzheimer’s disease

Alzheimer’s disease (AD) is the primary reason for dementia among people over the age of 65. It has two main pathological characteristics, one is senile plaques mainly composed by amyloid-β (Aβ) peptides which is resulted from aberrant amyloid precursor protein (APP) cleavage, and the other is neurofibrillary tangles (NFTs) basically consisting of paired helical filaments (PHFs) which are derived from hyperphosphorylated tau proteins (Lau and Ahlijanian, 2003). These two proteinopathies are the basic changes underlying the progressive neuronal degeneration and cognitive dysfunction in AD patients. In a population-based study, it was found that there was a correlation between the genetic variation of Cdk5 gene and the risk of AD (Arias-Vasquez et al., 2008). Elevated p25/p35 ratio was observed in multiple brain areas including frontal cortex, inferior parietal cortex and hippocampus in human AD brains (Tseng et al., 2002). P25 transgenic mouse model showed AD-like pathological changes such as tau hyperphosphorylation, NFTs, and neuronal loss. Transgenic expression of Cdk5 inhibitory peptide before and after the insult of p25 could both suppress hippocampal tau phosphorylation and neuronal death, improve the cognitive dysfunction of model mice (Xu et al., 2019; Huang et al., 2020). Inhibition of Cdk5/p25 complex also reduced hippocampal Aβ production and tau hyperphosphorylation, improved learning and memory abilities of AD model mice (Zhao et al., 2021).

Amyloid-β peptides are produced from the sequential cleavage of APP by β-amyloid cleavage enzyme 1 (BACE1) and γ-secretase. Aβ40 and Aβ42 are two main forms of Aβ peptide, the longer Aβ42 has a much stronger tendency to oligomerize and aggregate than the shorter Aβ40, and exhibits more neurotoxic effect. Accumulation of oligomerized Aβ peptides forms amyloid plaques, which is closely related with neuronal loss and cognitive decline in AD. Cdk5/p25 activity was upregulated and significantly correlated with BACE1 elevation in brains of AD patients and transgenic mouse models (Sadleir and Vassar, 2012). Wen et al. (2008) found that Cdk5 facilitated Aβ generation through modulation of BACE1 synthesis *via* signal transducer and activator of transcription 3 (STAT3), who binded to BACE1 promoter and phosphorylated it at Ser727. In cultured primary rat hippocampal neurons, Cdk5 activation triggered peroxisome proliferators-activated receptor γ (PPARγ) phosphorylation at Ser273, then increased BACE1 activity and Aβ production (Quan et al., 2019). Presenilin 1 gene mutation is the main reason for autosomal dominant familial forms of AD (Fraser et al., 2000). As the core subunit of γ-secretase, presenilin-1 (PS1) is essential for the catalytic activity of γ-secretase. Presenilin-1 dysfunction will result in the increase of Aβ42/Aβ40 ratio, which is more toxic than the rising of Aβ42 absolute amounts (Chavez-Gutierrez et al., 2012). Cdk5/p35 is reported to regulate PS1 stability and metabolism through phosphorylating PS1 at Thr354 (Lau et al., 2002), and the aberrant Cdk5 activity will cause γ-secretase dysfunction and the alteration of Aβ42/Aβ40 ratio.

Tau is a microtubule-associated protein primarily distributed in axons. Tauopathy is characterized by abnormal tau aggregation and NFT formation in the brain, which is a hallmark of AD. Cdk5/p25 can promote tau dimerization through phosphorylating it at different sites, which subsequently aggregate into PHF-like filament, resulting in microtubule collapse and neurite retraction (Paudel, 1997). The Cdk5 and hyperphosphorylated tau are found to be co-existed in the neocortical pyramidal neurons and cerebellar neurons of AD (Pei et al., 1998). Moreover, enhanced Cdk5 immunoreactivity was observed in neurons undergoing early stage of NFTs degeneration and plaque-neurites in several regions of human AD brain (Pei et al., 1998). Cdk5 hyperactivation was also found to induce the accumulation of hyperphosphorylated tau, Aβ plaques, and neuronal loss in animal models. TFP5, an inhibitory peptide of Cdk5/p25, significantly reduced tau hyperphosphorylation, neurofilament accumulation and restored synaptic function and behavior abnormalities in transgenic AD model mice as well as the mice overexpressing p25 through attenuating Cdk5 hyperactivity (Shukla et al., 2013, 2017). Long-term and short-term Cdk5 knockdown also both inhibited insoluble tau aggregation in the hippocampus of 3xTg-AD mice and improved their spatial memory (Castro-Alvarez et al., 2014). Collapsin response mediating protein-2 (CRMP2) is capable of promoting microtubule assembly through binding tubulin heterodimers (Fukata et al., 2002). It was reported that Cdk5 phosphorylated CRMP2 at Ser522 and reduced its binding to tubulin (Uchida et al., 2005). And this hyperphosphorylated CRMP2 was demonstrated to be a component of PHF (Yoshida et al., 1998), and were observed in the brain of AD transgenic mouse model (Cole et al., 2007). Cdk5 was also reported to activate microtubule affinity-regulating kinases 4 (MARK4) and increased tau phosphorylation and accumulation through increasing the phosphorylation of MARK4 at Ser262 (Saito et al., 2019).

Besides the above-mentioned Aβ generation and NFT formation, CNS inflammation caused by microglia activation is another main feature of AD, which correlates with the plaque accumulation and may exacerbate the pathology of AD (Kitazawa et al., 2005). Aβ oligomers caused long-lasting activation of microglia and profound neuroinflammation in the hippocampus, which was mediated by over-activation of Cdk5/p25 complex. Cdk5 inhibitor roscovitine suppressed the inflammatory processes evoked by Aβ (Wilkaniec et al., 2018). Increased phospholipase A2 (PLA2) activity is centrally involved in inflammatory responses associated with several neurological disorders including AD (Farooqui et al., 2006). Soluble lipid mediator lysophosphatidylcholine (LPC) is generated through PLA2-mediated phosphatidylcholine hydrolysis (Steinbrecher et al., 1984). It has been demonstrated that p25 overexpression lead to glia activation, neuroinflammation and neurodegeneration partially through cytosolic PLA2-mediated LPC release in neurons (Sundaram et al., 2012). In addition, hyperactivation of Cdk5 mediated by p25 accumulation also accounts for exacerbation of tau pathology caused by lipopolysaccharide, a toxin used to induce nervous system inflammation (Kitazawa et al., 2005).

Oxidative stress and dysfunctional mitochondria appear at the early stages of AD. Cdk5 is an upstream activator of mitochondrial dysfunction in AD. The mitochondrial damage results in more ROS generation and Ca^2+^ level elevation, which in turn lead to even higher Cdk5 activity, then shapes a vicious circle and contributes to neuron loss in AD (Sun et al., 2008). A variety of molecules and signaling pathways are involved in the oxidation caused by aberrant Cdk5. Sun et al. (2008) reported that over-activation of Cdk5 led to mitochondrial damage and ROS accumulation through inactivating Prx1 and Prx2. Drp1 is a direct target of Cdk5, and Cdk5-mediated phosphorylation of Drp1 at Serine 579 regulates Aβ1-42 induced mitochondrial fission and neuronal toxicity. Inhibition of Cdk5 attenuated Aβ1-42 induced mitochondrial fission by inhibiting Drp1 phosphorylation in primary cultured neurons (Guo et al., 2018). In cultured hippocampus HT-22 cells, Cdk5 inhibition also blocked mitochondrial fragmentation and AD-like hallmarks induced by streptozotocin through suppressing Drp1 phosphorylation (Park et al., 2020). JNK and p38 MAPK signaling pathways are able to be activated by oxidative stress and they are critically involved in Aβ-induced neurotoxicity and NFT formation (Yoshida et al., 2004). Enhanced JNK activity and increased p38 expression were observed in the affected brain areas of AD patients (Pei et al., 2001). Cdk5 interacts with p38/JNK pathways and contributes to the progressive Aβ deposits and AD development. In transgenic AD mouse model, co-immunoprecipitation of p-JNK and p-p38 with Cdk5 was significantly enhanced (Otth et al., 2003). Dysregulated Cdk5 caused elevation of p38 activity by increasing ROS in response to β-amyloid neurotoxic stimuli (Chang et al., 2010). As the major substrate involved in JNK-induced neurotoxicity, c-Jun is also over-activated in the several brain regions of AD patients. Cdk5 is reported to activate c-Jun through phosphorylating it at Ser63 and Ser73 *via* ROS-mediated activation of JNK (Sun et al., 2009). Myeloid cell leukemia sequence 1 (Mcl-1) is a member of the B-cell lymphoma 2 (Bcl-2) family, which is essential for neuronal survival (Arbour et al., 2008). The disease severity of AD patients was shown to be inversely correlated with Mcl-1 levels. Mcl-1 could be phosphorylated at Thr92 by Cdk5 following neurotoxic insults and this phosphorylation in turn caused Mcl-1 degradation and mitochondrial dysfunction, which promoted the neurodegenerative process of AD (Nikhil and Shah, 2017).

The aberrant Cdk5 activity seen in the progressive neurodegeneration of AD can also be caused by other mechanisms besides the dysfunction of its co-activators. Cancino et al. (2011) reported that Aβ-induced c-Abelson tyrosine kinase (c-Abl) activation facilitated tau phosphorylation *via* phosphorylating Cdk5 at Tyr15 *in vitro*. In the brains of AD mice, there was also an elevated Tyr15 phosphorylation of Cdk5 correlating with increased c-Abl levels (Cancino et al., 2011). Cdk5 could be S-nitrosylated by endogenously generated NO (Foster et al., 2009) and S-nitrosylated Cdk5 (SNO-Cdk5) in turn transnitrosylated Drp1 and resulted in mitochondrial fission in dendritic spines, which contributed to dendritic spine loss following Aβ treatment (Qu et al., 2011). Glutathione-S-transferase pi 1 (GSTP1) is able to dislodge p25/p35 and clear ROS accumulation and negatively regulates Cdk5 activity. There was a significant correlation between reduced GSTP1 levels and hyper-activation of Cdk5 in prefrontal cortex (PFC) of human AD brain (Sun et al., 2011). Furthermore, GSTP1 is capable of suppressing the activation of JNK/c-Jun pathway whereas it will lose its control following oxidative or chemical stress (Adler et al., 1999). GSTP1 was also shown to relieve the inhibition of Prx1 mediated by Cdk5 and reactivate it directly (Ralat et al., 2006).

**FIGURE 2:**
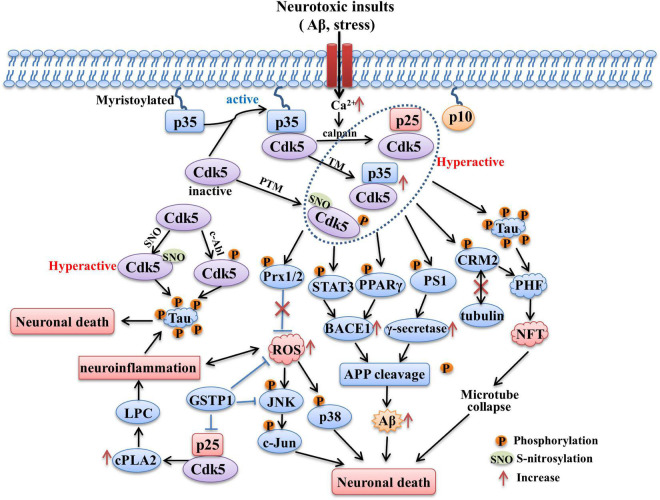
The role of Cdk5 in AD. Sustained activation of Cdk5 hyperphosphorylated tau protein and contributes to formation of PHF and NFT. CdK5 over-activation also induces intracellular Aβ accumulation through increasing APP processing mediated by BACE1 and γ-secretase. The phosphorylation of STAT3, PPARγ and PS1 contributed to the above process. Phosphorylation of Prx2 caused by aberrant Cdk5 activity inhibits its ROS scavenging ability and induces the activation of p38 MAPK and JNK pathways. Hyperactivation of Cdk5 also leads to neuroinflammation mediated by LPC accumulation through increasing cPLA2 activity. In addition, Cdk5 activity could be upregulated through being S-nitrosylated by endogenously generated NO and phosphorylated by c-Abl, both of process contribute to tau hyperphosphorylation. The tau hyperphosphorylation, NFT formation, intracellular Aβ generation, oxidative stress and neuroinflammation caused by aberrant Cdk5 activity all contribute to the neuronal death and neurodegeneration in AD.

The summation of above molecular pathways related with Cdk5 in AD is presented in Figure 2.

### The role of cyclin-dependent kinases 5 in Huntington’s disease

Huntington’s disease (HD) is a progressive neurodegenerative disease characterized by chorea, personality changes and dementia (Kaminosono et al., 2008). It is mainly caused by selective loss of striatal medium-sized spiny neurons, resulted from abnormal polyglutamine (polyQ) expansion in the protein huntingtin (htt) (MacDonald et al., 2003). The role of Cdk5 and its co-activators in HD is still controversial. Some studies have reported their neuroprotective effects, while others have shown the opposite results.

It has been demonstrated that htt co-localized with Cdk5 in cellular membrane (Luo et al., 2005). Cdk5 could phosphorylate htt at Ser1181 and Ser1201 both in primary striatal cultures and in the mouse striatum following DNA damage, which prevented neuronal death and exerted neuroprotective effects. In the brain of HD mouse model, decreased Cdk5/p35 association at the late stage resulting from sustained DNA damage led to htt dephosphorylation and polyQ-induced p53 mediated neuronal death (Anne et al., 2007). Low levels of Cdk5 and p35 have been found in the striatum of postmortem HD patients and in HdhQ111 mutant mice (Luo et al., 2005; Paoletti et al., 2008). Consistent with this, there was an accumulation of p25 and increased p25/p35 ration in rat striatum following delivery of quinolinic acid (QA) (Park et al., 2012), a neurotoxin used to make experimental model of HD.

Phosphorylation of huntingtin at Ser421 has been shown to be neuroprotective against intrastriatal QA injection (Metzler et al., 2010). In contrary to the above results, Park et al. (2017) found that single p35 allele deletion resulted in a 17% enhancement of htt phosphorylation at Ser421, indicating that genetic reduction of p35 may be neuroprotective in the context of HD. Moreover, p35 hemizygous knockout mice also showed a much smaller striatal lesion size induced by QA compared with control (Park et al., 2012). Cherubini et al. (2015) reported that the increased activity and translocation of Drp1 to the mitochondria mediated by Cdk5 contributed to striatal neurodegeneration in HD. Inhibition of Cdk5 activity could attenuate mitochondrial fragmentation in striatal cells through modulating Drp1 activity and subcellular location. Cdk5 was also shown to be involved in the hippocampal and corticostriatal-dependent learning and memory impairment relevant to HD. Genetic reduction of Cdk5 alleviated corticostriatal-dependent learning deficits by increasing GluN2B surface levels in the cortex and striatum of HD model mice. Cdk5 knockdown enhanced Rac1 activity and subsequently increased hippocampal dendritic spine density, which may be the mechanism underlying the improvement of hippocampal-dependent memory (Alvarez-Periel et al., 2018).

These findings suggest the multifaceted roles of Cdk5 in HD. Future studies are still needed to further clarify the relationship between Cdk5 dysfunction and HD pathogenesis.

### Cyclin-dependent kinases 5 dysfunction contributes to stroke

Stoke is an acute neurologic event caused by dramatic reduction of cerebral blood flow to a localized part of brain (Bacigaluppi and Hermann, 2008). According to the affected brain regions, the cerebral ischemia can be divided into focal and global ischemia stroke (Traystman, 2003). A study analyzed the causes of death in Chinese residents from 1990 to 2017 and surprisingly found that stroke ranked the first, not cancer (Zhou M. et al., 2019). Many patients are disabled or died because of the untimely treatment due to the narrow time window of thrombolytic therapy. In addition, quite a few patients are not suitable for thrombolysis. Therefore, it is still necessary to further identify the pathological mechanisms underlying stroke and search for the potential pharmacological targets.

Disruption of the tight regulation of Cdk5 has been observed in stoke-affected brain tissue of patients (Mitsios et al., 2007), as well as in animal and cellular models of stroke (Meyer et al., 2014). The analysis of Cdk5 expression in human post-mortem brain showed that Cdk5- and p-Cdk5-positive neurons and microvessels increased dramatically in stroke-affected regions, accompanied by irregular arrangement and clumped in the cytoplasm (Mitsios et al., 2007). Ischemia induced either by oxygen glucose deprivation (OGD) in brain slices or middle cerebral artery occlusion (MCAO) *in vivo* both induced the cleavage of p35 into p25 (Meyer et al., 2014). Pharmacological inhibition or Cdk5 knockdown using the approach of peptide-directed lysosomal degradation prevented neuronal death, reduced infarction size and promoted the recovery of neurological functions following MCAO in mice (Meyer et al., 2014; Zhou Y. F. et al., 2019). In rat hypoxia/ischemia (HI) injury model, Cdk5 activity and p25 level were also significantly increased, and inhibiting Cdk5 with small peptide reduced cerebral infarct volume and promoted functional recovery (Tan et al., 2015).

The mitochondrial dysfunction and oxidative stress are also critically involved in the pathogenesis of stroke. Mitochondrial fusion and fission are two important ways to regulate mitochondrial function, balanced fusion/fission is essential for maintaining normal cellular physiology and fusion/fission imbalance will cause mitochondrial dysfunction and degeneration. Drp1 is a GTPase that controls the process of mitochondrial fission, and the elevated Drp1 phosphorylation will cause excessive mitochondrial fission. Hyper-activation of Cdk5 could increase the phosphorylation of Drp1 at Ser616, which in turn increased GTPase activity and accelerated mitochondrial fission, ultimately contributed to mitochondrial dysfunction and neuronal death following OGDR insults (Chen et al., 2021). Inhibition of Cdk5 hyperactivity could attenuate mitochondrial fragmentation and neuronal loss through reducing Drp1 phosphorylation at Ser616 (Chen et al., 2021). In human body, the oxidative/antioxidative balance is kept by a large group of antioxidant enzymes under physiological condition, which can catalyze hydrogen peroxide into stable non-toxic molecules (Apel and Hirt, 2004). Prx2 is such an antioxidant enzyme. Cdk5 phosphorylates Prx2 at Thr89 and this phosphorylation decreases Prx2 peroxidase activity and lowers its capacity to eliminate ROS which contributes to neuron loss subsequently (Qu et al., 2007). In both focal and global ischemia, elevated phosphorylation of Prx2 at Thr89 and ROS accumulation were found due to upregulated cytoplasmic Cdk5 activity (Rashidian et al., 2009). And p35 knockout mice displayed remarkably smaller infarct size following stroke insult due to a lower level of Prx2 phosphorylation (Rashidian et al., 2009).

The dysfunction of *N*-methyl-D-aspartate receptors (NMDARs) contributed to a variety of neurological diseases including stroke. Phosphorylation of NMDAR subunits at the cytoplasmic carboxyl termini is an important way to regulate NMDAR function. It has been shown that Cdk5 can phosphorylate NMDAR at multiple sites. The phosphorylation of GluN2A at Ser1232 by Cdk5 enhanced receptor function and promoted CA1 pyramidal neuron death following transient forebrain ischemia (Wang et al., 2003). The phosphorylation of GluN2B at Tyr1472 by Cdk5 promoted receptor internalization (Zhang et al., 2008), similarly phosphorylation of regulatory site Ser1116 also inhibited the membrane trafficking of GluN2B-containing NMDARs (Plattner et al., 2014). Cdk5 was also shown to phosphorylate GluN2B at Ser1284, which may contribute to the ischemic injury in both cultured neurons suffered from OGD-reperfusion and mice subjected to transient global ischemia (Lu et al., 2015). The anaphase-promoting complex/cyclosome (APC/C) is a E3 ubiquitin ligase that controls cell cycle progression (Peters, 2002). Cdh1 is the activator of APC/C and cyclin B1 is one substrate of APC/C–Cdh1, whose nuclear level is increased in affected neurons of stroke patients (Love, 2003). It is well known that abnormal release of glutamate caused by NMDAR overactivation is involved in stroke (Bossy-Wetzel et al., 2004). Cdk5 contributes to NMDAR-mediated neuronal excitotoxicity by phosphorylating Cdh1 at residues Ser40, Thr121, and Ser163, resulting in Cdh1 inactivation and cyclinB1 accumulation in the nucleus (Maestre et al., 2008).

Cyclin-dependent kinases 5 can modulate the process of cell apoptosis and cell cycle through regulating the function of transcription-related molecules, which may be another mechanism involved in stroke. MEF2 is a factor critical for neuronal survival. Activating MEF2 is pro-survival, whereas hyperphosphorylation of MEF2 contributes to neuronal apoptosis (Mao and Wiedmann, 1999). MEF2 can be phosphorylated at Ser408 and Ser444 directly by Cdk5 and this phosphorylation will lead to reduced MEF2 function due to the inhibition of MEF2 transactivation activity (Gong et al., 2003). Nuclear Cdk5 contributes to neuronal death following focal ischemia but not global ischemia by phosphorylating MEF2 (Tang et al., 2005). Histone deacetylase 1 (HDAC1) is critically involved in suppressing transcription of cell cycle genes and regulating the cell cycle (Lagger et al., 2002). Cdk5 was shown to inhibit HDAC1 function and induce double-strand DNA breaks and cell cycle reentry, resulting in neurodegeneration and neurologic defects following ischemia (Kim et al., 2008).

The Cdk5-modulated pathways involved in stroke are shown in Figure 1.

### Cyclin-dependent kinases 5 dysfunction triggers anxiety/depression

Anxiety is the emotional response to threatening or potentially threatening stimuli (Sandford et al., 2000) and it is beneficial for the animals to cope with the stress. However, excessive anxious state or pathological anxiety is harmful, characterized by hypervigilance to threatening stimuli or negative events (Rosen and Schulkin, 1998). Depression is mainly manifested by anhedonia, loss of motivation and abnormal neurovegetative functions (Nestler and Carlezon, 2006). Anxiety and depression have become major public health issues and bring multiple challenges to the society. The current clinical strategies for depression and anxiety mainly act through modulating serotonergic system or GABAergic system. However, these medical approaches often have inadequate efficacy and can’t meet clinical needs. Therefore, an urgent need to find new potential therapeutic targets emerges.

More and more evidences have shown that Cdk5 dysfunction plays important roles in psychiatric disorders. Analyzing postmortem brains of patients with major depression showed that Cdk5 activity was significantly increased in Brodmann’s area 25, a subregion of the PFC, which was implicated in major depression and treatment response (Papadopoulou et al., 2015). In rat depressive model induced by chronic mild stress, Cdk5 activity and p35 trafficking was enhanced in the hippocampal dentate gyrus, which was suppressed by the antidepressant venlafaxine (Zhu et al., 2012). The elevation of Cdk5 activity was also observed in several brain regions of anxious mice including basolateral amygdala, septal and lateral septum (Bignante et al., 2008). Pharmacological inhibition or genetic knockout of Cdk5 both significantly attenuated the depressive-like symptoms or aberrant anxiety-like phenotype in mice (Li G. et al., 2014).

There are multiple mechanisms involved in the regulation of anxiety/depression by Cdk5. The dysfunction of glucocorticoid receptor (GR) signaling critically contributed to the pathology of psychiatric disorders. Cdk5 was shown to phosphorylate GR at serine 232 and there was a significant correlation between elevated Cdk5 activity and enhanced GR phosphorylation in hippocampus and PFC of the depressive rats (Mitic et al., 2013). The mechanism study revealed that the aberrant GR phosphorylation may be attributed to the dysfunction of Cav1.2 subunit of the L-type calcium channel, which was closely related with stress-induced neuropsychiatric conditions. Lower levels of p25 production and GR phosphorylation was observed in the Cav1.2 heterozygous (Cav1.2^ + ⁣/−^) mice compared with control mice following chronic unpredictable stress, correlating with reduced depressive-like and anxiety-like behaviors (Bavley et al., 2017). Depression is the most common psychiatric comorbidity of HD. Inhibition of Cdk5 activity in the nucleus accumbens attenuated the depressive-like behaviors of HD mice through modulating dendritic spine plasticity mediated by dopamine- and cAMP-regulated phosphoprotein 32 (DARPP32)/β-adducin signaling pathway (Brito et al., 2019). Anomalous neurotransmission is an important mechanism underlying anxiety disorder. Cdk5 was shown to regulate anxiety-like behaviors through modulating neurotransmission and neuronal excitability. Conditional deletion of Cdk5 in parvalbumin (PV) interneurons results in increased GABAergic neurotransmission which in turn alleviated the anxiety-like behavior of mice (Rudenko et al., 2015). Optogenetic study showed that Cdk5 activation led to the decreased activation of excitatory neurons in the prelimbic cortex, and Cdk5 knockdown reversed the deactivation of these excitatory neurons and alleviated the anxiety-like behaviors induced by chronic inflammation (Wang et al., 2015).

### Cyclin-dependent kinases 5 dysfunction elicits pathological pain

Acute nociceptive pain is the physiological sensation of injury that helps animals survive by promoting them to withdrawal from the harmful stimuli and avoid further contacting with such stimuli. However, pathological and chronic pain seems meaningless and will cause much distress. Allodynia and hyperalgesia are two kinds of behavioral phenotypes indicated by decreased threshold or amplification in the responsiveness to noxious stimulation following chronic pain (Rahn et al., 2013). Mounting evidences have shown that Cdk5 is a key molecule involved in pain modulation. In mice model of peripheral inflammatory pain, there were both an elevation of Cdk5 activity in dorsal root ganglia (DRG) and spinal dorsal horn (Wang et al., 2005; Yang et al., 2007). Roscovitine, a Cdk5 inhibitor, alleviated heat hyperalgesia induced by CFA and formalin in a dose-dependent manner (Wang et al., 2005). Conditional deletion of p35 in mice (p35-/-) resulted in decreased sensitivity to painful thermal stimulation due to reduced Cdk5 activity, whereas mice overexpressing p35 exhibited thermal hyperalgesia (Pareek et al., 2006).

Cyclin-dependent kinases 5 participates in the inflammatory pain-induced hypersensitivity through diverse pathways. Extracellular signal-regulated kinase 1 and 2 (ERK1/2) is fast activated in response to innocuous and noxious stimulation (Ji et al., 2002; Cruz et al., 2005). The levels of phosphorylated ERK1/2 (p-ERK) and phosphorylated Cdk5 at Ser159 (p-Cdk5) are increased in spinal cord dorsal horn following adjuvant-mediated inflammatory pain (Zhang X. et al., 2014). And inhibition of ERK1/2 significantly suppresses the nociceptive responses and enhancement of p-ERK and p-Cdk5 (Zhang X. et al., 2014). Tumor necrosis factor-α (TNF-α), a proinflammatory cytokines, participates in the onset and development of inflammatory pain. The release of TNF-α is elevated following peripheral inflammation, which triggers the activation of ERK1/2 and then results in the induction of early growth response 1 (Egr-1), a member of zinc-finger *trans*-activators. Subsequently, Egr-1 binds to the promoter region of p35 and increases Cdk5 activity (Utreras et al., 2009). These results indicate that ERK-mediated Cdk5 activation plays a vital role in the hypersensitivity of peripheral inflammatory pain.

Transient receptor potential vanilloid 1 (TRPV1) is mainly expressed in the nociceptive sensory neurons and potentiate pain sensitization in different pain models (Caterina et al., 1997; Davis et al., 2000). The phosphorylation of TRPV1 plays an important role in responding to a pain stimulus by regulating intracellular calcium levels (Pareek et al., 2007). Cdk5 regulates TRPV1 membrane trafficking through phosphorylating TRPV1 at the site of Thr407. Conditional Cdk5 knockout in small diameter sensory neurons (C-fibers) abolishes TRPV1 phosphorylation and leads to hypoalgesia (Pareek et al., 2006, 2007). Transforming growth factor-β1 (TGF-β1) signaling pathway is involved in modulating Cdk5-mediated TRPV1 phosphorylation under the condition of inflammation pain. TGF-β1 treatment increased Cdk5-mediated phosphorylation of TRPV1 at Thr407 *in vitro*. The conditional TGF-β1 knockout reduced Cdk5 activity and Cdk5-dependent TRPV1 phosphorylation with attenuation of thermal hyperalgesia in mice following inflammatory pain (Utreras et al., 2012). TNF-α was shown to increase p35 expression, causing Cdk5-mediated TRPV1 phosphorylation and ROS production in nociceptive neurons and increased pain sensation (Sandoval et al., 2018). These results indicate that TRPV1 and TGF-β signaling participate in the modulation of pain sensation by Cdk5. BDNF-tyrosine kinase, type 2 (TrkB) signaling pathway has been shown to be critically involved in pain modulation. TrkB activation contributes to initiation and maintenance of both heat and mechanical hypersensitivity produced by tissue injury (Wang et al., 2009). Cdk5 is demonstrated to phosphorylate and activate TrkB at Ser478 residue (Lai et al., 2012). Inhibiting Cdk5 attenuates CFA-induced hypersensitivity through suppressing TrkB expression and blocking BDNF/TrkB signaling pathway, implying that the interaction between Cdk5 and BDNF/TrkB also plays a role in pain hypersensitivity induced by inflammation (Zhang H. H. et al., 2014).

Besides its involvement in inflammatory pain, Cdk5 has been also shown to modulate several other kinds of pain such as neuropathic pain (Li K. et al., 2014; Yang et al., 2014), bone cancer pain (Zhang R. et al., 2012), visceral pain (Chang et al., 2011), post-operative pain (Liu et al., 2014), orofacial pain (Prochazkova et al., 2013; Hu et al., 2022). For example, in the mouse neuropathic pain model induced by spinal nerve ligation, the expression of Cdk5 and its activators in DRG was elevated, which contributed to the mechanical allodynia (Gomez et al., 2020). Cdk5 inhibition attenuated mechanical allodynia and thermal hyperalgesia through suppressing mGluRs and (or) NMDAR phosphorylation in both bone cancer pain (Zhang R. et al., 2012) and post-operative pain (Liu et al., 2014) models. Depletion or overexpression of p35 caused mice to be less or more sensitive to orofacial mechanical stimulation, respectively (Prochazkova et al., 2013). Inhibition of Cdk5 activity could also alleviate facial pain by suppressing calcium-mediated trigeminal peripheral sensory neurons activation (Hu et al., 2022). The crucial roles of Cdk5 in pain modulation make it a promising non-opioid target for pain treatment.

### Cyclin-dependent kinases 5 dysfunction contributes to epilepsy

Epilepsy affects over 70 million people worldwide, which is mainly manifested by recurrent seizures and brings a variety of physiological and psychosocial consequences (Thijs et al., 2019). The levels of Cdk5, p-Cdk5 and its kinase activity were significantly increased in the anterior temporal lobe samples from the patients with mesial temporal lobe epilepsy accompanied by hippocampal sclerosis (Banerjee et al., 2021). Hippocampal sclerosis (HS), characterized by segmental neuronal loss and gliosis, is the most common cause of refractory epilepsy in adults. The ratio of p25 to p35 and activity of Cdk5/p25 complex increased remarkably in the diseased hippocampi compared with the adjacent normal temporal lobe, indicating a pathological role of Cdk5/p25 in HS (Sen et al., 2006).

Sustained endoplasmic reticulum (ER) stress triggers the regional specific astroglial responses, which contributed to the status epilepticus (SE). Cdk5 phosphorylation was shown to be upregulated in the astrocytes within the hippocampus CA1 region and dentate gyrus following ER stress. Inhibition of Cdk5 with roscovitine markedly suppressed hippocampal astroglial response induced by ER stress (Lee and Kim, 2021), which may be mediated by reducing PKA activity and Drp1 phosphorylations (Hyun et al., 2017). Roscovitine was also shown to suppress SE-induced neuroinflammation mediated by glial responses *via* p38 MAPK inhibition in rat frontoparietal cortex (Kim et al., 2019). Blood–brain barrier (BBB) dysfunction is critically involved in epilepsy. Specific deletion of Cdk5 in the endothelial cells of BBB resulted in spontaneous seizures in mice, which may be attributed to the decreased astrocytic glutamate reuptake and elevated glutamatergic synaptic function (Liu et al., 2020).

## Cyclin-dependent kinases 5 substrates

As one up-stream kinase in the nervous system, Cdk5 triggers the down-stream signaling pathways through interacting with its substrates. Numerous Cdk5 substrates have been detected and their abnormal alterations following aberrant Cdk5 activity are closely related with initiation and progression of neurological disorders. In this review, some important substrates of Cdk5 are listed in Table 1. The readers can be referred to prior reviews with a more comprehensive list of Cdk5 substrates (Dhavan and Tsai, 2001; Su and Tsai, 2011; Pao and Tsai, 2021).

**TABLE 1:** List of major Cdk5 substrates reviewed in neurological disorders.

Substrates	Phosphorylation sites	Outcome	References
Drp1	Ser616 and Ser579	Promoting mitochondrial fission	Cherubini et al., 2015; Guo et al., 2018; Park et al., 2019; Chen et al., 2021
Parkin	Ser131	Reducing its E3 ubiquitin-ligase activity	Avraham et al., 2007
Glycoprotein 78	Ser516	Ubiquitination and degradation	Wang et al., 2018
Peroxidases 2	Thr89	Reducing its peroxidase activity and inducing ROS accumulation	Qu et al., 2007; Sun et al., 2008; Rashidian et al., 2009
EndoB1	Thr145	Autophagy induction	Wong et al., 2011
MEF2	Ser408 and Ser444	Inhibition of MEF2 transactivation activity and neuronal death	Gong et al., 2003; Zhang Q. et al., 2016
RKIP	Thr42	Cell cycle re-entry and neuron death	Wen et al., 2014
PPARγ	Ser273	Promoting BACE1 activity and Aβ production	Quan et al., 2019
Presenilin-1	Thr354	Dysfunction of γ-secretase and the alteration of Aβ42/Aβ40 ratio.	Lau et al., 2002
Tau	Multiple sites	Microtubule collapse and neurite retraction	Paudel, 1997
CRMP2	Ser522	Reducing microtubule assembly	Uchida et al., 2005
MARK4	Ser262	Tau phosphorylation and accumulation	Saito et al., 2019
Peroxidases 1	Thr90	Mitochondrial damage and ROS accumulation	Sun et al., 2008
c-Jun	Ser63 and Ser73	Activation of JNK pathway	Sun et al., 2009
Mcl-1	Thr92	Mcl-1 degradation and mitochondrial dysfunction	Nikhil and Shah, 2017
Huntingtin	Ser1181, Ser1201, and Ser421	Reducing neuronal death	Anne et al., 2007; Metzler et al., 2010
GluN2A	Ser1232	Enhancing receptor function	Wang et al., 2003
GluN2B	Tyr1472 and Ser1116	Promoting receptor internalization	Zhang et al., 2008; Plattner et al., 2014
Cdh1	Ser40, Thr121, and Ser163	CyclinB1 accumulation and neurotoxicity	Maestre et al., 2008
Glucocorticoid receptor	serine 232	Depressive phenotype	Mitic et al., 2013
TRPV1	threonine 407	Hyperalgesia	Pareek et al., 2007
TrkB	Ser478	Allodynia and hyperalgesia	Lai et al., 2012; Zhang X. et al., 2014

## Conclusion

**FIGURE 3:**
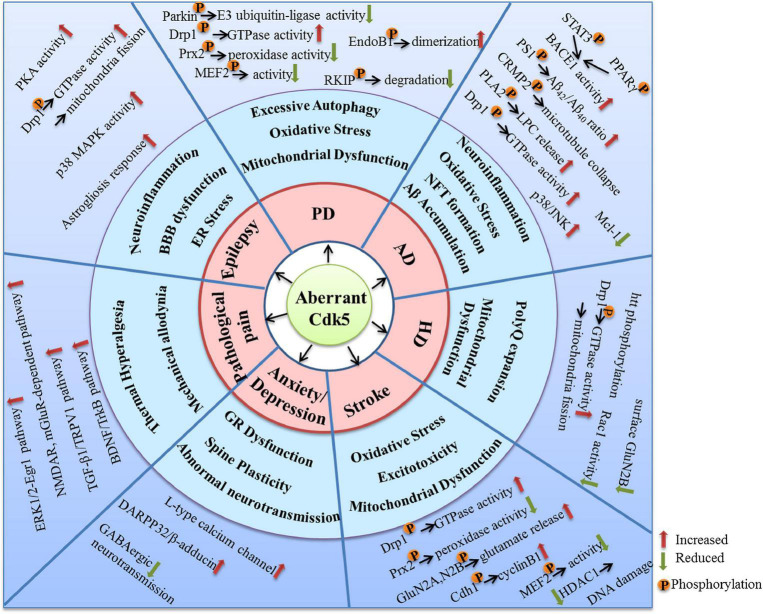
The molecular targets and mechanisms involved in the neurological disorders caused by aberrant Cdk5 activity.

As a kinase distributed extensively in the nervous system, Cdk5 plays crucial roles in various kinds of neuronal function. Aberrant activation of Cdk5 appears to be a driving force for the initiation and progression of multiple neurological disorders. Numerous molecular targets and pharmacological mechanisms are involved in the Cdk5-mediated neurodysfunction (Figure 3). The extensive actions of Cdk5 discussed in this review suggest that it is a promising therapeutic target for neurological diseases. In fact, many small-molecule inhibitors and peptides have been synthetized and their beneficial effects in nervous system have been examined. Although quite a few inhibitors exhibit good neuroprotective properties in various preclinical studies, no selective Cdk5 kinase inhibitors have ever entered clinical trials for therapeutic intervention. A successful Cdk5 inhibitor for neurological diseases should be able to pass the BBB and have a high Cdk5 selectivity. Non-selective Cdk inhibitors are generally unfavorable and ineffective due to the potential off-target toxic and side effects. For example, roscovitine, one of the best known Cdk inhibitors, have been shown promising protective effects in nervous system through inhibiting Cdk5. However, roscovitine can also inhibit Cdk1, Cdk2, Cdk7, and Cdk9, the low selectivity for Cdk5 reduces its potential as a drug candidate targeting neurological disorders.

As the aberrant Cdk5 activity is generally caused by p25 accumulation, so small molecule protein-protein interaction (PPI) inhibitors targeted to disturb the interactions between Cdk5 and p25 may represent a promising strategy. It can not only eliminate Cdk5/p25 complex-induced hyper-activation but also retain the physiological function of Cdk5/p35. In addition, there is no concern about the selectivity, as the inhibitors are developed to target the specific PPI site of Cdk5 and p25 without affecting other Cdks kinase activity. However, it can be predicted that the development of any kind of drugs, including the small molecule PPI inhibitors, will not always be smooth. But it may shed light upon the treatment of Cdk5-related neurological diseases. Future extensive studies are still needed to define more precisely the role of Cdk5 hyperactivation in brain function impairments, and it will facilitate the discovery of more effective therapeutic strategies targeting Cdk5 aberrant activity implicated in neurological disorders.

## Author contributions

ZT and JT designed the review. JT and X-QW searched the literature. ZT and BF made the figures and table. ZT, X-QW, BF, and JT wrote and edited the original draft. All authors have read and agreed to the published version of the manuscript.
